# Agrivoltaic systems in a tropical region: Influence on thermal environment, vaginal temperature, and behavior of dairy heifers

**DOI:** 10.3168/jdsc.2025-0971

**Published:** 2026-04-23

**Authors:** Elen Kaline Sartori, Maria José Hötzel, Karolini Tenffen De-Sousa, Teresa Cristina Alves, Matheus Deniz

**Affiliations:** 1 Programa de Pós-Graduação em Agroecossistemas, Universidade Federal de Santa Catarina, 88062-000, Florianópolis, Santa Catarina, Brazil; 2 Laboratório de Etologia Aplicada e Bem-Estar Animal, Universidade Federal de Santa Catarina, 88062-000, Florianópolis, Santa Catarina, Brazil; 3 Grupo de Estudos em Bovinos Leiteiros, Faculdade de Medicina Veterinária e Zootecnia, Universidade Estadual Paulista, 18610-034, Botucatu, São Paulo, Brazil; 4 Empresa Brasileira de Pesquisa Agropecuária, Embrapa Pecuária Sudeste, 13560-970, São Carlos, São Paulo, Brazil

## Abstract

This study aimed to evaluate the influence of different agrivoltaic system configurations on the thermal environment, vaginal temperature, and behavior of dairy heifers raised on pasture in a tropical climate. The study was carried out between July and August 2025, covering the winter season in the Southern Hemisphere. Microclimatic variables were measured, and the black globe-humidity index (BGHI) was calculated to determine thermal comfort. Additionally, vaginal temperature and behavior of 21 dairy heifers were measured in a crossover experimental design and compared among 3 treatments: an agrivoltaic system with panels installed in a vertical position (AGRIver), an agrivoltaic system with panels installed in a horizontal position (AGRIhor), and an open pasture system (OPS). All data were analyzed by mixed generalized linear models, and the results are presented using as estimated marginal means (emmeans ± SE). Both agrivoltaic systems (AGRIver and AGRIhor) showed lower values for the microclimatic variables and BGHI compared with the OPS, except for relative humidity. Treatments did not influence the heifers' vaginal temperature (AGRIver: 38.5°C ± 0.046°C; AGRIhor: 38.67°C ± 0.045°C; OPS: 38.71°C ± 0.046°C). Animals were observed more frequently standing ruminating in the OPS, and were observed most frequently lying in the agrivoltaic systems (AGRIver: lying rumination; AGRIhor: lying idle). Heifers visited the water trough more frequently in the AGRIhor system (emmeans ± SE; 20.2 ± 4.35 events/h) and tended to visit it less frequently in the AGRIver system (14.7 ± 3.19 events/h) compared with the OPS (16.5 ± 3.57 events/h). In OPS, heifers engaged more frequently in affiliative behaviors (3.92 ± 0.40 events/h) compared with agrivoltaic systems (AGRIver: 2.91 ± 0.32 events/h; AGRIhor: 3.15 ± 0.34 events/h). Furthermore, heifers interacted more frequently with photovoltaic panel structures in AGRIhor (4.02 ± 0.91 events/h) than in AGRIver (2.41 ± 0.56 events/h). In conclusion, agrivoltaic systems provided a better thermal environment for dairy heifers and influenced lying behaviors (rumination and idle) compared with the open pasture. Additionally, heifers used the photovoltaic panel structures as environmental enrichment.

## Highlights

• Agrivoltaic systems reduced microclimatic heat load compared with an open pasture system.
• Heifers' vaginal temperature increased with time of day but did not differ among treatments.
• Heifers performed more lying behaviors and affiliative interactions in agrivoltaic systems.
• Heifers interacted more frequently with photovoltaic panel structure in the horizontal position.

## Graphical Abstract

Solar photovoltaic power plants occupy large areas, making it challenging to find suitable locations for large installations. One solution to this problem is to integrate photovoltaic systems into farmland. We evaluated how 2 agrivoltaic system configurations, horizontal and vertical photovoltaic panels, shaped the thermal environment, vaginal temperature, and behavior of dairy heifers raised on pasture. Both systems reduced heat load, providing more comfortable conditions than open pasture. Beyond offering shade, the panel structures were also used by the animals to perform scratching behavior. The compact and wider shade created by the vertical panels likely supported more frequent lying rumination, whereas the narrower and longer shade created by the horizontal panels allowed more space for scratching behavior.

**Figure:**
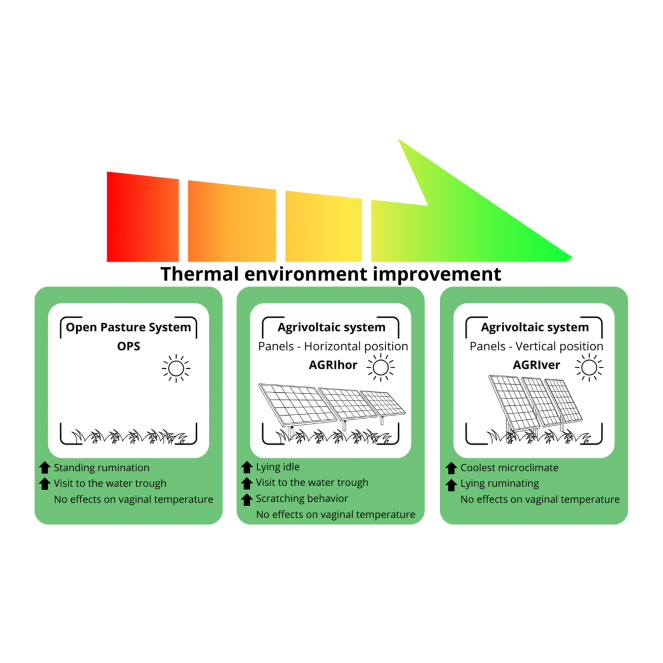

Providing shade (natural: Pezzopane et al., 2019; De-Sousa et al., 2023; or artificial: Schütz et al., 2010) is essential to improving animals' thermal comfort in pasture-based systems. Moreover, it influences the behavior and physiology of cattle (Deniz et al., 2023). Agrivoltaic systems (i.e., systems that integrate photovoltaic panels with crops or animals in the same area simultaneously) are gaining prominence in the context of emerging technologies and increasing investments in clean energy production (Manito et al., 2026).

Introducing photovoltaic panels in pasture areas allows for the integration of shade provision to improve the thermal comfort of animals, as well as reducing carbon emissions on farms (Maia et al., 2020) and improving land use (Dupraz et al., 2011). Although the effect of photovoltaic panels on heat load, behavior, and physiology of farming animals (sheep: Maia et al., 2020, and cattle: Faria et al., 2023; Sharpe et al., 2021) is attracting the attention of the scientific community, research involving livestock farming in agrivoltaic systems is still scarce (Bacon et al., 2025; Manito et al., 2026). For example, there is no information on the influence of the configuration of photovoltaic panels on dairy cattle behavioral and physiological responses. Thus, the objective of this study was to evaluate the influence of different agrivoltaic system configurations compared with an open pasture system on the thermal environment, vaginal temperature, and behavior of dairy heifers raised on pasture in a tropical climate.

The study was carried out between July and August 2025 (covering the winter season of Southern Hemisphere) at the agrivoltaic system of the “Centro Avançado de Pesquisa, Inovação e Desenvolvimento de Bovinos Leiteiros,” at the Lageado Experimental Farm of the School of Veterinary Medicine and Animal Science at the São Paulo State University (Botucatu, São Paulo state, Southeast Brazil). The climate of the region is characterized as Cwa under the Köppen climate classification (Alvares et al., 2013), with hot and rainy summers and dry and cold winters, with 2 well-deﬁned seasons: a dry season (April to September, average temperature of 18.3°C) and a rainy season (October to March, average temperature 22.5°C). Annual accumulated precipitation is ~1,500 mm, spread over 107 d, on average.

During the experiment, heifers were kept permanently on pasture. The pasture area was composed of 24 paddocks (2,000 m^2^) managed under a rotational grazing system (Pinheiro Machado Filho et al., 2021), and each paddock was equipped with one plastic water trough (500 L). Among these paddocks, the experimental area was composed of 9 paddocks of 2,000 m^2^ each: 3 open pasture system (**OPS**) paddocks, representing traditional grazing conditions, located adjacent to the agrivoltaic systems (**AVS**) area and used as control; and 6 AVS paddocks equipped with 72 photovoltaic panels measuring 2.28 × 1.14 m, with a power capacity of 580 Wp and a maximum efficiency of 22.45%. In 3 paddocks, 12 panels/paddock were installed in a vertical position (**AGRIver**), and in the remaining 3 paddocks, 12 panels/paddock were installed in a horizontal position (**AGRIhor**). The panels were installed side by side (without spacing) in the center of each paddock, east–west orientation and facing north, at a fixed height of 2.30 m and a 10° slope, to provide 24 m^2^ (3.4m^2^ shade/animal) of useful shaded area. In AGRIver, the shaded area was more compact and wider (12 m length × 2 m width), whereas in AGRIhor, it was narrower and longer (24 m length × 1 m width). The OPS paddocks contained only pasture, without any shading structures. The experiment was carried out during the dry season (April–September). In all paddocks, the pasture was predominantly composed of *Brachiaria decumbens*. The pasture had an approximate height of 30 cm and an average of 605.20 kg DM/ha (7.8% CP; 65.3% NDF/DM) and was managed uniformly across the 3 systems (OPS, AGRIver, and AGRIhor). Pasture availability was approximately the same in all the 3 systems (OPS, AGRIver, and AGRIhor) and reflected typical regional conditions, with equal pasture growth observed among systems. Given the season, low pasture availability was expected; therefore, all animals received supplementation twice a day (0800 h and 1700 h) with corn silage (42% DM) and mash commercial feed (16% CP).

The experimental design was adapted from the methodology proposed by Schütz et al. (2009). Twenty-one crossbred (Holstein × Jersey) dairy heifers (average ± SD, 16.4 ± 2.5 mo old and weight of 275.8 ± 42.1 kg) were divided into 3 groups of 7 heifers/group in a crossover experimental design. For animal-related variables (behavior and vaginal temperature), the experimental unit was the group of animals (n = 7), as treatments were applied at the group level. All heifers had previous experience with the 3 pasture systems and were habituated to being handled in groups and to the presence of observers for 2 consecutive days before the data collections. The groups were balanced for similar age and body mass (group 1 = 16.1 ± 2.4 mo and 275.4 ± 49.9 kg; group 2 = 16.7 ± 2.6 mo and 275.6 ± 40.3 kg; group 3 = 16.2 ± 2.7 mo and 276.4 ± 42.2 kg) and rotated through the 3 pasture systems (OPS, AGRIver, and AGRIhor), remaining in each paddock for 2 consecutive days (2 d/pasture system).

The experimental period was selected based on the weather forecast, as cloudless days were a requirement to measure the intensity of animals' use of the different areas of the systems. All heifers were exposed to all treatments twice, resulting in a replicated experimental period lasting 12 d in total. Data were collected on all 12 d of the experimental period, and the 3 heifer groups were always observed simultaneously on the same days, ensuring comparable climatic conditions across treatments. Data (microclimatic variables, vaginal temperature and behavior) were collected between 9 h and 16 h.

The following microclimate variables recorded were collected every 5 min by 5 set autonomous dataloggers (for details and validation, refer to Deniz et al., 2022): air temperature (**AT**; °C), relative humidity (**RH**; %), soil surface temperature (**SST**; °C), and black globe temperature (**BGT**; °C). The AT, RH, and BGT were measured 1.3 m above the soil, and SST at the soil below the pasture. In the AVS, one datalogger was located at the shaded condition provided by the photovoltaic panels and the other at full sun exposure (sunny condition). From the microclimatic data, the black globe-humidity index (**BGHI**) was calculated using the equation proposed by Buffington et al. (1981), and BGHI >74 is considered a threshold value for the start of thermal stress in dairy cattle (Baêta and Souza, 2010). For microclimate variables, the experimental unit was the paddock, as measurements were recorded at the paddock level.

The vaginal temperature was recorded using a centi-T logger (accuracy: ± 0.1°C, resolution: ± 0.032°C; Star-Oddi, Gardabaer, Iceland), configured to record data every 5 min, and attached to a shortened, hormone-free controlled internal drug release (CIDR, GlobalGen Vet Science, Jaboticabal, São Paulo, Brazil) using heat-shrink tubing (Tresoldi et al., 2020). The behavior events (grazing, eating, ruminating, idle; Coimbra et al., 2012) and posture (standing or lying) were recorded directly by scan-sampling at 10-min intervals (Altmann, 1974). The number of water intake events (animal with the lips immersed in the water, with neck movements indicating water ingestion; Coimbra et al., 2012), affiliative interactions (repeated tongue movements by the performing animal in contact with the body of another animal; Agudelo et al., 2013), and interactions with photovoltaic panel structures (animals scratching any part of their body on the photovoltaic panel structures) were recorded continuously whenever they occurred (Altmann, 1974; Lehner, 1996). Behaviors were always recorded by 2 groups of 3 observers trained by K.T.S. and M.D. Because the 3 groups were observed simultaneously during the data collection period, each observer observed only one group at a time to ensure accuracy and consistency in data collection. To assess interobserver reliability, all observers recorded behaviors in live sessions for 2 h across a minimum of 2 d (before the beginning of the data collection), and reliability values were taken from these. All possible behaviors were exhibited in the training session, and the observers were trained until they reached reliability ≥90% (Cohen's kappa, Gamer et al., 2019), compared with K.T.S. and M.D.

All data were analyzed in R (R version 4.4.2; R Core Team, 2024) with the aid of RStudio, using mixed generalized linear models (**GLM**). The models were adjusted through the maximum likelihood-Laplace approximation method in the statistical package lme4 (Bates et al., 2015). In all models, the normality of residuals was evaluated through graphic inspection. The Akaike information criterion was used to evaluate the model fit. After fitting the model, estimated marginal means were obtained using the emmeans package (Lenth, 2017) and means were compared through the Tukey test (*P* < 0.05). Confidence intervals were estimated using type II Wald chi-squared tests, and the fit of the model was given by a likelihood test. The model's significant effects were defined as *P* < 0.05, and a tendency at *P* < 0.10. For interpretation purposes, results are presented as estimated marginal means (**emmeans** ± SE) obtained from the fitted models, which account for the fixed and random effects specified in each analysis. To confirm that treatments (OPS, AGRIver, and AGRIhor) and areas (shaded and sunny) influenced the microclimate variables and the BGHI, data were summarized by hour, using a GLM with Gaussian distribution (95% CI). The model consisted of treatments as fixed effects, with hours nested within day as a random effect to correct for day-specific conditions that may influence time budgets. To assess the influence of treatments on vaginal temperature, the data were summarized by hour, using a GLM with Gaussian distribution (95% CI). The model consisted of treatments and hours (continuous variable) as fixed effects, heifer groups and days as random effects. To confirm that the treatments influenced heifers' behavior, the data were analyzed by a mixed GLM with Poisson distribution (95% CI). The models consisted of treatments as fixed effects, with heifer groups and hours nested within day as a random effect.

Treatments influenced (*P* < 0.05) the microclimatic variables and thermal comfort indicators. Both AVS (AGRIver and AGRIhor) showed lowest values for the microclimatic variables and BGHI (Table 1) compared with the OPS, except for RH.

**Table 1:** Estimated means ± SEM obtained from the regression models with the Gaussian distribution and 95% CI of the variables air temperature (AT), relative humidity (RH), soil surface temperature (SST), black globe temperature (BGT), and black globe-humidity index (BGHI) in the different treatments during the 12 d of data collection (9–16 h)^1^

Variable	Pasture system						*P*-value
	AGRIver		AGRIhor		OPS		
	Average	SEM	Average	SEM	Average	SEM	
AT (°C)	22.10^c^	0.99	22.50^b^	0.99	23.00^a^	0.97	<0.001
RH (%)	50.40^b^	3.29	58.30^a^	3.29	48.70^c^	3.29	<0.001
SST (°C)	20.00^c^	1.20	23.10^b^	1.19	28.00^a^	1.20	<0.001
BGT (°C)	24.41^c^	1.17	25.32^b^	1.17	28.81^a^	1.19	<0.001
BGHI	70.10^c^	0.98	71.60^b^	0.98	74.60^a^	0.99	<0.001

a–c Mean values in a row followed by the same letter do not differ.

1 Treatments: agrivoltaic system with vertical panels (AGRIver), agrivoltaic system with horizontal panels (AGRIhor), and open pasture system (OPS).

Within the AVS treatments, lowest values (*P* < 0.001) of AT (emmeans ± SE; AGRIver shaded: 21.5°C ± 0.84°C, AGRIver sunny: 22.9°C ± 0.84°C; AGRIhor shaded: 21.8°C ± 0.81°C, AGRIhor sunny: 23.3°C ± 0.83°C), SST (AGRIver shaded: 17.2°C ± 0.89°C, AGRIver sunny: 24.9°C ± 0.95°C; AGRIhor shaded: 18.9°C ± 0.95°C, AGRIhor sunny: 27.3°C ± 0.95°C), and BGHI (AGRIver shaded: 67.6 ± 1.11, AGRIver sunny: 73.8 ± 1.13; AGRIhor shaded: 69.0 ± 1.08, AGRIhor sunny: 74.1 ± 1.08) were observed in the shaded areas. In contrast, the RH was higher (*P* = 0.0004) in the shaded areas (AGRIver: 53.8% ± 2.37%; AGRIhor: 59.2% ± 2.13%) than in the sunny areas (AGRIver: 47.9% ± 2.37%; AGRIhor: 57.3% ± 2.16%).

Treatments did not influence (*P* > 0.05) heifers' vaginal temperature. In the OPS, average (emmeans ± SE) heifers' vaginal temperature was 38.71°C ± 0.046°C (range: 38.55°C–38.84°C). In the AGRIver, heifers' average vaginal temperature was 38.5°C ± 0.046 (range: 38.54°C–38.84°C; *P* = 0.726). In the AGRIhor, heifers' average vaginal temperature was 38.67°C ± 0.045°C (range: 38.52°C–38.82°C; *P* = 0.127). Regardless of treatment, time of day influenced the heifers' vaginal temperature. Within the experimental period (range: 9 h–16 h) for each 1-h increase, average vaginal temperature increased 0.082°C (β = 0.082 ± 0.004; t = 20.76; *P* < 0.001).

The budget of behaviors across all treatments (OPS, AGRIver, and AGRIhor) is shown in Table 2. Grazing was the predominant activity, regardless of treatment. Standing rumination behavior was highest in the OPS and AGRIhor, whereas lying rumination was highest in the AGRIver and lying idle was highest in the AGRIhor. Furthermore, heifers visited the water trough more frequently (*P* = 0.001) in the AGRIhor system (emmeans ± SE; 20.2 ± 4.35 events/h) and tended to visit it less frequently (*P* = 0.09) in the AGRIver system (14.7 ± 3.19 events/h) than in the OPS (16.5 ± 3.57 events/h).

**Table 2:** Estimated means ± SEM of the number of events obtained by hours of observations per day from the regression models with the Poisson distribution and 95% CI of behaviors performed by group of heifers (7 heifers/group) in the different treatments during the 12 d of data collection (9–16 h)^1^

Behavior	AGRIver		AGRIhor		OPS	
	Average	SEM	Average	SEM	Average	SEM
Grazing	9.52^a^	0.69	9.42^a^	0.69	9.06^a^	0.66
Eating	3.41^a^	0.71	2.86^a^	0.59	3.08^a^	0.64
Lying rumination	1.93^a^	0.85	1.58^b^	0.69	1.63^b^	0.72
Standing rumination	0.54^b^	0.21	0.63^ab^	0.24	0.73^a^	0.28
Lying idle	0.73^c^	0.31	1.17^a^	0.49	1.02^b^	0.43
Standing idle	0.61^a^	0.08	0.77^a^	0.09	0.68^a^	0.09

a–c Mean values in a row followed by the same letter do not differ.

1 The estimated means correspond to the expected number of events per hour (events/h). Treatments: agrivoltaic system with vertical panels (AGRIver), agrivoltaic system with horizontal panels (AGRIhor), and open pasture system (OPS).

In the OPS, heifers engaged more frequently (emmeans ± SE: 3.92 ± 0.40 events/h) in affiliative behavior compared with the AVS (AGRIver: 2.91 ± 0.32 events/h; *P* = 0.045; AGRIhor: 3.15 ± 0.34 events/h; *P* = 0.046). No difference (*P* = 0.72) was found between the 2 AVS. However, heifers interacted more frequently (*P* < 0.001) with photovoltaic panel structures in AGRIhor (emmeans ± SE: 4.02 ± 0.91 events/h) than in AGRIver (2.41 ± 0.56 events/h).

Our results showed that AVS provided a better thermal environment for dairy heifers than OPS, although thermal conditions varied among the different agrivoltaic system configurations. The AGRIver presented the lowest values of AT, SST, and BGHI. Previous studies showed that shade from solar panels reduces heat load (Sharpe et al., 2021; Carvalho Fonsêca et al., 2023; Faria et al., 2023), but no study has evaluated different agrivoltaic system configurations. The influence of shade configuration has been studied in silvopastoral systems, showing that variations in tree density, species, and spatial arrangement significantly influence the microclimate (Pezzopane et al., 2019; De-Sousa et al., 2021). Although AVS are an emerging technology with the potential to be used as an alternative source of shade in pasture-based systems (Bacon et al., 2025), there is a gap in knowledge regarding the effect of different solar panel configurations. Future studies should explore how panel configuration influences the thermal environment and animal thermal comfort.

The absence of an effect of the treatments on the vaginal temperature of the heifers suggests that under our experimental conditions, the thermal environment was not sufficiently challenging to modify the circadian body temperature pattern of the heifers. Similarly, Palacio et al. (2015) did not find differences in the body temperature of cows with or without access to shade during mild weather. However, studies conducted in thermal challenging conditions have shown that the shade provided by photovoltaic panels (Sharpe et al., 2021; Faria et al., 2023) was effective in reducing the animals' body temperature. Overall, these findings reinforce the conclusion that the impact of AVS on animals' body temperature depends on the intensity of the thermal challenge to which the animals are exposed and the specific metabolic heat production according to physiological stage (e.g., lactating, nonlactating, and pregnant cows).

The animals' behavior differed among treatments. Standing rumination was more frequent in the OPS and AGRIhor. Normally, standing behaviors are more likely to occur in areas without shade or in areas where the quality of the shade is limited (Deniz et al., 2023), as they represent a behavioral strategy that aids in heat dissipation (Allen et al., 2015). Cattle adopt different behavioral strategies to maintain homeostasis, and the thermal environment (e.g., solar radiation: Magalhães et al., 2020; AT: Volpi et al., 2021; and SST: De-Sousa et al., 2021) markedly influences animal behavior. On the other hand, the lowest values of BGHI and SST may have contributed to the highest frequency of lying rumination in AGRIver. Even though the shaded areas are similar in both AVS, the geometry of these areas plays an important role in microclimatic quality. More compact shaded areas provide a lower perimeter to area ratio, present fewer exposed edges, thereby reducing the incidence of solar radiation. So, shade geometry can directly influence the solar radiation incidence and enthalpy of air, affecting the animals' capacity to dissipate heat and maintain thermal homeostasis (de Castro Júnior and Silva, 2021). In the AGRIver system, the wider and more compact shade likely promoted the formation of a central shade nucleus where the incidence of direct and lateral radiation was lower (mainly during periods of low solar angle, such as in the morning and late afternoon). In contrast, in narrower and longer shaded areas, such as AGRIhor, this effect may be smaller. Although this environment may have offered a less favorable microclimate than AGRIver, it still represented an improvement over OPS, as indicated by the highest frequency of lying idle in AGRIhor. However, this less favorable microclimate may have been reflected in the higher frequency at the water trough in the AGRIhor and OPS system. It has also been shown that sheep can spend less time at the water through when kept in AVS than in open pasture (Andrew et al., 2021), highlighting the need for greater research effort on the effects of AVS configurations in dairy cattle behavior.

Heifers engaged more frequently in affiliative behavior in the OPS, whereas in the AVS, they used the panel structures to scratch on. Our results contrast with Améndola et al. (2016) who found that animals express more socio-positive behaviors in pasture with shaded areas (e.g., silvopastoral systems) compared with treeless pasture. In our study, the heifers likely exhibited more affiliative behaviors in the OPS than in the AVS because there were no structures to interact with in the OPS. The contrast between studies may be explained by differences in resource availability rather than a contradiction in animal welfare outcomes. In both cases, animals appear to adaptively express motivated behaviors depending on environmental opportunities. Both behaviors are indicative of positive animal welfare, whereas affiliative behavior involves interactions between 2 animals and has been associated with the promotion of herd cohesion (Reinhardt and Reinhardt, 1981), scratching behavior is a self-directed activity that promotes individual comfort, in which the animal scratches parts of its body to clean the coat (McConnachie et al., 2018). Cattle are motivated to use abrasive surfaces to scratch on, such as trees in silvopastoral system (De-Sousa et al., 2021). Thus, providing opportunities for cattle to perform this behavior can help them cope with environmental stressors (Newby et al., 2013), improving their welfare (McConnachie et al., 2018). To our knowledge, this is the first study to describe how cattle use panel structures to scratch on. Therefore, future research in AVS should include scratching behavior as an indicator of animal welfare and assess the potential risks and benefits this behavior may pose to the animals, as well as the longevity of photovoltaic installations.

In conclusion, the AVS provided a better thermal environment for dairy heifers than the OPS. The lack of an effect on vaginal temperature can be explained by the season in which the study was conducted. The AVS also influenced heifers' behavior. The wider and compact configuration of AGRIver provided the coolest microclimate, which was reflected in the greater number of observations of lying rumination. The narrower and longer configuration of AGRIhor allowed more space for scratching behavior.
